# Hadoop in Banking: Event-Driven Performance Evaluation

**DOI:** 10.1155/tswj/4375194

**Published:** 2025-01-21

**Authors:** Monalisa Panda, Mamata Garnayak, Mitrabinda Ray, Smita Rath, Anuradha Mohanta, Sushree Bibhuprada B. Priyadarshini

**Affiliations:** ^1^Department of Computer Science and Engineering, Siksha ‘O' Anusandhan Deemed to be University, Bhubaneswar, India; ^2^Department of Computer Science, Kalinga Institute of Social Sciences, Deemed to be University, Bhubaneswar, Odisha, India; ^3^Department of Computer Science and Information Technology, Siksha ‘O' Anusandhan Deemed to be University, Bhubaneswar, India

**Keywords:** card-analytics, distributed Hadoop-file-system, event-analytics, event-log, financial report format, Hadoop architecture, Hive query, log file

## Abstract

In today's data-intensive atmosphere, performance evaluation in the banking industry depends on timely and accurate insights, leading to better decision making and operational efficiency. Traditional methods for assessing bank performance often need to be improved to handle the volume, velocity, and variety of data generated in real time. This study proposes an event-driven approach for performance evaluation in banking alongside a Hadoop-based architecture. Infused with real-time event analytics, this scalable framework can process and analyze fast-moving transactional data. Hence, the framework allows banks to monitor key performance indicators and detect real-time operational anomalies. This is supported by the Hadoop ecosystem, which provides distribution of the processing and storage, making it fit for handling large datasets with high fault tolerance and parallel computation levels. This study analyzes transaction and user engagement data using Hive queries, focusing on credit card transactions via MasterCard. Two cases are examined: a detailed snapshot of individual transactions and a five-day trend analysis. Metrics like active users, card registrations, and retention are visualized through dashboards. Findings reveal user activity patterns and areas for improvement, emphasizing scalable, data-driven approaches for transaction analytics. This framework conceives a functional approach for banks to exploit extensive data-analytic capabilities to strive for competitive advantage and survivability of a business by adding any required metrics. The findings signify that the Hadoop-integrated event-driven analytics method could act as a game changer for performance evaluation in the banking sector.

## 1. Introduction

Over time, the Indian banking industry has reached a standalone height. The operational patterns of banks [1–3] have been fundamentally reshaped by the adoption of advanced technologies and the rapid expansion of data. Users and researchers can achieve reasonable long-term profitability with a proactive approach accompanied with regular, trustworthy, and short-term financial reports. Without understanding the organization's financial fluency, it is not easy to describe its thriving [4, 5]. Therefore, it is of the utmost importance to monitor the organization's financial affairs in terms of required report formats in greater depth. It includes a variety of performance metrics for the banking industry, such as the number of registered users, active cards, and transaction details, among other essential things, in addition to an effort to use Hadoop architecture to improve marketing and customer service management [6, 7] for the current voluminous amount of data. Regarding digitalization and intellectualization of society, the current half of the 20th century has been characterized by computerization and all areas of financial activity. Similarly, regarding integrated large collaborative systems, a reasonable increase in human activity enhances the device count tied with the modeled architectures [8].

The fast-growing data today, in structured, semistructured, and unstructured forms, hold valuable insights. Traditional tools often struggle to extract insights from vast, diverse data sources. Big data analytics provides an efficient solution by abstracting meaningful information from raw, voluminous data. To address bank issues effectively, we utilized system-generated test case data from an industry-based project, demonstrating the power of big data analytics in banking event analysis.

According to Rawat, big data analytics provides a powerful solution by bringing out the key features that respond to the challenges posed by data, namely, issues inherent in managing its scale, speed, and complexity [9]. Hariri, Fredericks, and Bowers emphasize that the rapid growth of emerging technologies continuously puts big data analytics on the frontline of artificial intelligence, management, and governance. Sun, in 2019, opines, “A small data strategy can complement big data analytics while effectively addressing the issues of big data” [10].

Since the economic reforms of 1991–1992, the banking industry began adopting computer-based systems in a large way to cater to a broader range of operations regarding its growing activities [11]. Digitally transformed in recent years, card payment has become one of the most widely utilized methods for performing online banking transactions, owing mainly to its speed and convenience [12–15].

“Events” in such analytics refer to changes in the states of network devices captured mostly as data points whenever a client enters a page or system. On analyzing, these events can provide insight into customer preferences, especially about banking transactions involving cards. By considering the volume and velocity of event data, Hadoop architecture provides an excellent way to design for storing, processing, and analyzing event-based information. The impetus of this study is to use Hadoop to formulate predictions on customer behavior patterns in banking for enhanced decision making and business acumen. Henceforth, we are motivated to use Hadoop architecture to identify and comprehend customers' preferences regarding various card-related activities in the financial sector.

Based on the generated reports of daily, weekly, and monthly, this research enhances future forecasts, which could assist in predicting and comprehending various events. Financial institutions will be able to make stronger speculations, allowing them to periodically improve their business based on probabilities and predictions of the resulting data.

The rest of the paper is divided into various sections. A comprehensive background study of the research is included in Section 2, specifically focusing on various financial reports.  Section 3 explains the related research. Problem formulation and proposed solution strategy are covered in Sections 4 and 5. The experimental simulation is included in Section 6, and the paper is concluded in Section 7.

## 2. Background Study

Clear and concise financial dashboards provide precise, aggregated visualizations of a company's financial data. These dashboards offer an accurate overview of the company's event analysis over specific periods, supporting informed decision making and future planning. Monthly or daily financial report analysis can be used to predict performance measures, giving you an advantage over your competitors. It includes the company's goals by weighing the benefits and drawbacks of the collected data and the associated financial goals. These estimates will precisely show their industry's year-over-year profit and growth. According to a recent study, we can accumulate opaque financial reports to conceal the news for an extended period [16].

The fundamentals of such report types are the primary focus of our research, which is illustrated below.

### 2.1. Monthly Financial Report

A monthly report shows a keen and thorough profit–loss statement incorporated through the detailed transactional category, respective data, card types, and precise information. It gives a detailed, concise, and up-to-date visualization of monthly cash flows [17]. Through this report, all the essential and necessary metrics can be drilled down over a four-week period, which can make a comprehensive comparison of financial affairs to other monthly reports of the year [18].

### 2.2. Weekly Report

This type of report aids in monitoring various short-term activities of the company every week, which is gradually merged to generate a monthly report. These reports are prepared weekly and thoroughly examined to provide a comprehensive short-term look at the business's performance.

*Cash flow report*: The cash flow report is incorporated by tracking various cash flows that reflect the exact visualization of the cash transfer during a durable period.

*Operational activities*: This report incorporates a company's operating cash movement by performing the net sum on the relevant metric measurement.

*Activities for financing*: It tracks any internal stock purchases or payments made in cash level changes.

*Activities for investing*: The particulars of cash changes about any kind of long-term investment are the focus of these activities.

We have examined a variety of daily, weekly, and monthly report templates specifically related to the financial sector of all businesses so far. Along with these, one must take the necessary steps to comprehend a clear financial visualization that comprehensively explains their particular financial application domain. These reports can be viewed in greater depth, giving insight into the following areas.

*Management of cash*: a comprehensive and in-depth look at the company's current cash flow situation.

*Loss and gain*: a look at the company's income in the most important areas, as shown by the loss and profit statement.

*The overall picture*: The format of the daily, weekly, and monthly reports provides a comprehensive financial activity overview to facilitate the creation of long-term strategies for the company's faster growth and increased profitability.

### 2.3. Optimizing Banking Performance With Event-Driven Reports

Monthly financial reports, weekly performance reports, and cash flows are critical in strengthening the event-driven performance evaluation model, especially when integrated into a Hadoop-based architecture in the banking sector. Here is a brief description of how such reports can contribute to the model.

i. *Data aggregation and trend analysis*: Monthly reports synthesize trends in revenue, profitability, and expenses, using Hadoop to compare real-time data with historical benchmarks and visualize long-term performance trends.
ii. *Anomaly detection*: Monthly data help detect deviations from expected performance, enabling early identification and mitigation of financial risks.
iii. *Short-term monitoring*: Weekly reports capture recent performance changes, allowing the model to detect high-frequency issues that may go unnoticed in monthly reports.
iv. *Operational efficiency insight*: Weekly data highlight bottlenecks or inefficiencies, like increased transaction times, allowing for quick corrective actions and improved operational performance.
v. *Liquidity and risk management*: Cash flow reports provide critical insights into liquidity and cash positions. By integrating real-time cash flow data with Hadoop, the model detects shifts in inflows or outflows, aiding in risk management.
vi. *Event-based anomaly detection*: Cash flow data help identify irregular transaction patterns, detecting financial stress, fraud, or unusual activities. Event analytics in Hadoop ensures compliance with regulatory standards.

Integrating reports into the event-driven model:

• *Benchmarking*: The model sets live benchmarks for key performance indicators (KPIs) like transaction volume and cash flow stability.
• *Dynamic decision making*: The model quickly adapts to new data, providing real-time recommendations based on trends to keep the bank responsive to changes.
• *Data-driven insights*: Big data analytics refine the model's ability to uncover deeper insights, improving the accuracy of performance assessments.

Overall, the reports provide past, trend-oriented, operational data to add impetus to the event-driven model's predictive and diagnostic power and facilitate a more comprehensive and timely assessment of the bank's performance.

## 3. Related Work

In information technology (IT), any change in the state of a network component is specifically referred to as an “event”. Every IT device produces events as “log entries” throughout a typical operation. This is stored in various repositories and databases as “event data.” The management of these event data is the primary focus of event analytics. To the best of our knowledge, a few works have been done in the analysis of banking data by applying Hadoop architecture. Therefore, in this section, we aim to provide a comprehensive review by including studies from various dimensions within this domain.

Gidwani et al. say that [19] event analytics conducts additional statistical analysis of events to avoid any accidental incidents or risks. The authors of this study used the prescribed algorithms to create a knowledge database for their subsequent development. The best way to describe the motion of this object is to use the story of an “event,” which Yan has explained in [20] by critically comparing significant elements of a surveillance event.

Through a cloud server, the system with a wireless sensor network can provide remote data [21]. In a research [22], the authors have provided an event monitoring survey by event-driven wireless sensor networks for event detection and reliable event information dissemination. Here, dissemination protocols for event information are presented and contrasted as the primary design concerns regarding event detection. Finally, a few issues that need to be resolved are listed. Nandgaonkar, Hanchate, and Deshmukh provided an overview of various information retrieval methods like “Topic Detection and Tracking,” “event tracking,” and “event evolution” approaches [23]. To demonstrate the relationship between events, they plotted an event evolution graph. Ryan and others [24] defined “Events 4.0” and investigated the digitized event maturity using the “Industry 4.0” model. They conducted a quantitative tourism survey and social media analysis using various methods.

“Event management” is a higher phase of “event analytics” that integrates multiple systems into one platform to identify underlying issues. With machine learning, cloud, and universal analytics, organizations gain a competitive edge. Effective big data use requires collecting relevant data from various sources.

We can have a more significant impact on our intended audience with more data. Big data technologies make it possible for intelligent city systems to evaluate the city on a micro level [25, 26]. Twitter data about London are studied using machine learning and big data to identify the spatiotemporal events in London. Over three million tweets were analyzed to arrive at the presented results [27–29]. The researchers of a study offered a conceptual model to convert extensive datasets into useful information and formulated a framework for transforming data into actionable knowledge [30, 31].

This survey highlights the role of event analytics in IT, where big data and machine learning drive proactive decision making and risk prevention. By utilizing diverse data sources, organizations like banking sectors can enhance performance and maintain a competitive advantage in the digital era. Hadoop may cause undue delays in handling most immediate user requirements in performing various transactions. Scaling it to accommodate further growth in banking data creates a higher complexity in maintaining the system and makes it harder to scale and maintain.

## 4. Problem Formulation

**Figure 1:**
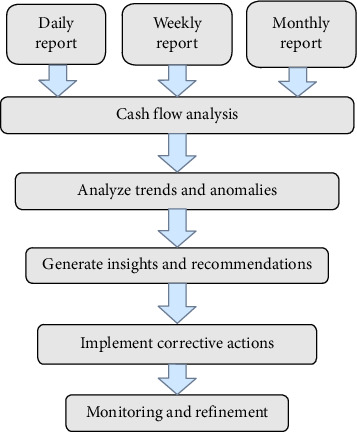
Workflow for financial report integration and analysis.

To meet the growing demand for digital banking services and overcome existing challenges, we focus on enhancing operational efficiency by optimizing banking transactions through an improved user interface in our proposed model. This model reduces inefficiencies by ensuring consistent data, accurate data capture, and automation, ultimately improving banking operations.  Figure 1 illustrates the process flow of integrating daily, weekly, and monthly financial reports into a Hadoop-based system for trend analysis, anomaly detection, insight generation, and continuous monitoring. As far as we know, this work is the first of its kind to apply such a comprehensive framework for banking data analysis.

In summary, the model aims to

i. Generate accurate daily, weekly, monthly, and annual data on user registrations and card activities.
ii. Track active user counts and measure business performance based on user engagement across various timeframes.
iii. Monitor the number of registered, active, and removed cards, including specific metrics for MasterCard, Visa, and RuPay issuers.
iv. Identify and address issues such as unsuccessful card registrations and pending card applications to enhance system reliability.
v. Analyze user retention rates weekly, monthly, and annually to assess application engagement and business growth.
vi. Provide actionable insights to reduce system inefficiencies, detect anomalies, and improve user satisfaction.

In order to clarify the difficulties above and effectively capture the necessary data, we define a few events in the systems. Within the proposed model, these events will be recorded alongside the day-to-day customer interactions, providing a consolidated view of those interactions.

We propose a practical solution approach that is integrated through the Hadoop server that will accelerate the banker's team to monitor their business performance by making use of the prescribed Hive queries. The product team can use the same strategy to ensure that the system is bug-free and increase its strength and competitiveness in the current market.

## 5. Proposed Solution Strategy

**Figure 2:**
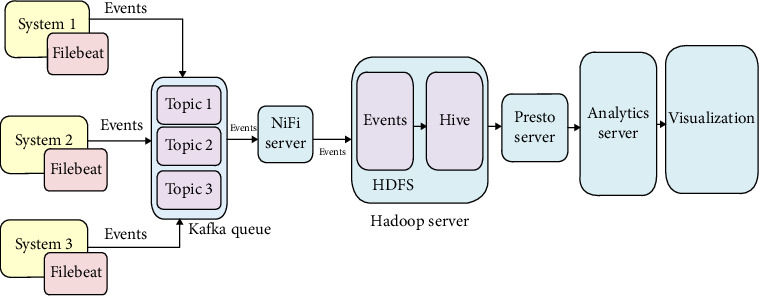
Modeled Hadoop server architecture.

We use a technology stack consisting of Hadoop Distributed File System—(HDFS, Hive), Kafka, Presto, NiFi, and Java to clarify and capture the data to overcome the above above challenges. Our solution approach consists of several events processed through the modeled Hadoop architecture. The detailed depiction of the proposed architecture (Hadoop server) is characterized in Figure 2.

Figure 2 depicts the enterprise's applications, which runs and ships the logs that pass through various architectural units as defined in the architecture. These units are (i) log shipper, (ii) messaging queue, (iii) intermediate storage, (iv) permanent storage, (v) analytics server, and (vi) visualization unit. The defined units are focused on generating various log events from several systems. System 1, System 2, etc. are the servers that house the enterprise application servers. Depending on the application, these systems produce various log events. Filebeat monitors and sends these log files to the messaging queue.

When a banking transaction is referred to as an “Event,” it alters the account balance in various ways. It is a transaction status action that takes place at a specific time. Our current approach's events are summarized in Table 1 with the appropriate abbreviations.

When a third-party transaction is carried out, a number of log events are recorded. The possible log events at various timestamps are included in Table 1.

The “Kafka service” serves as the messaging queue in this case. Its primary function is to assemble data from available servers and deliver it to the “NiFi server” through a pipeline. There is no data loss or duplication, even if the Kafka server goes down somehow. On the other hand, Kafka restores each bit of event data without any division.

NiFi's job is to separate and store events in the “HDFS” according to the date and application type (day/month/year). It is a robust and integrated platform for data logistics that can automate data movement between disparate systems.

**Figure 3:**
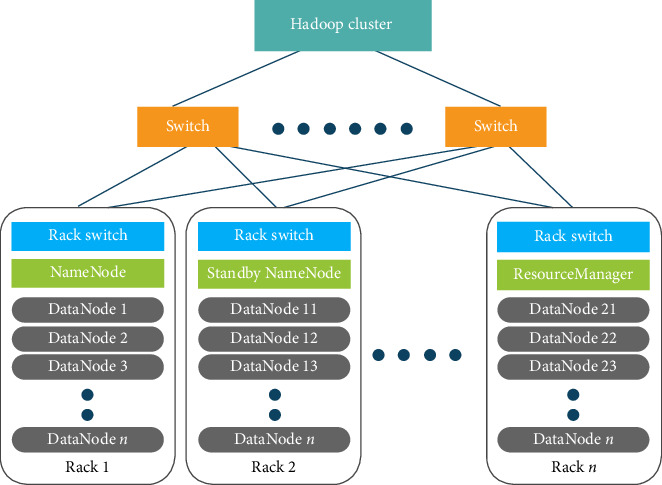
Architecture of “Hadoop cluster.”

The log-based data storage system known as the “Hadoop cluster” is highly available as shown in Figure 3. There are several “data nodes (where the actual data gets stored)” distributed across “*n*” racks in the architecture. The various “switches” connecting these racks are responsible for transferring the input data by availability. In order to make it simple to back up data if they are lost, the storage of data is based on data replication. In the suggested architecture, we employed a three-replication factor. It indicates that the similar data are replicated in three different nodes in three different racks. Hence, we can guarantee high data availability in this manner.

In a similar vein, there are several nodes designated as “name node,” “standby name-node,” and “resource manager,” which make it easier to locate the necessary data from the appropriate data node. To apply query processing to retrieve the relevant data, the “name-node” component identifies the required data nodes. The “standby name-node” and “resource manager” are brought in to perform the necessary data retrieval if the name-node fails to retrieve.

From gigabytes to petabytes, “Presto” is a distributed SQL query engine that runs analytical queries. It allows analytics over a large area and can combine data from multiple sources.

Again, the “analytics server” is a web server with few services running. The data are obtained from the Hadoop system by them. Then, the same data are processed using a specific procedural language called “Hive queries,” which is very similar to SQL and is used to query, monitor, and manage larger databases.

The “JavaScript Object Notation Format (JSON)” generates the final analytics data. The generated data are presented in a variety of formats, including daily, weekly, monthly, and annual. When accessed through a browser, the visualization service connects to the analytics server and presents the JSON data as a table.

### 5.1. Generated Event Tables

We will be able to generate data on a daily, weekly, monthly, and annual basis using several performance metrics that will be visible in a table using the above architecture. The following is a list of the metrics our system considers.

• Users who have registered: The particular metric can learn how many users are registered in the system daily, weekly, monthly, and yearly.
• Active user count: The metric reflects the active users on a daily, monthly, weekly, and yearly basis, allowing us to verify the performance of the business generated.
• The number of user cards registered: This metric reveals the number of users registered with the system.
• The number of cards in use: The measurement mirrors the quantity of proactive cards that are not lapsed in the framework.
• The number of registered cards: Regardless of the card issuer, this metric displays the total number of cards registered in the system.
• A total number of MasterCard holders: It indicates the number of users and cards associated with the issuer “MASTERCARD.”
• The number of Visa cards in use: It focuses on determining the number of users and cards associated with the issuer “VISA.”
• The number of enrolled RuPay cards: The metric determines how many people have signed up for the “RuPay card” issuer.
• Number of unsuccessful card registrations: The product team mostly uses this metric to check the system's robustness and bug-freeness. This is to keep track of the system's growth and number of failures.
• The total number of cards awaiting registration: The product team will be able to see how many cards are pending through this metric. This could be a problem with our system, the card issuer section, or any other cause that can be fixed once it is found.
• The number of cards that were removed: The business team can use this metric to see how many cards have been removed daily, weekly, monthly, and annually, providing a clear picture of the company's business situation.
• The number of users who were kept for the past week: This metric is the number of unique users who used the application, completed a transaction, and are still active this week. It is a weekly report.
• Monthly retention rate of users: This shows the number of unique users who used the application and made a purchase and how many of those users are still active in the current month. It is like a monthly report.
• Retained users from the previous year: This is a yearly metric that shows the number of unique application users since the previous year.

### 5.2. Event Pseudocode

The current section contains a sample of an event where the user logs into the system to start a transaction. The following event will initiate the transaction and contain all user information. Unique request ID, application version ID, event type, event duration, status information, and card and device information are some of these kinds of information.

Event sample:

  “requestId”:ca45dt-qur56e-8uyd76-jsT5t6; //“Unique Request ID”//
  “appVersion”: 2.0.0.6;//“Application Version”//
  “appId”: Tpay;//“Application ID”//
  “userId”: 9902496905;//“User Id”//
  “eventTYpe”: TPCLTRNSINI//“TransactionInitiation”//
  “status”: SUCCESS;//“Event status”//
  “eventTime”: 2021-19-04 19:13:21.579;//Event Time//
  “statusCode”:200;//“Event status code”//
  “traceId”: 12674;//“Trace an event”//
  “deviceModel”: Samsung-lf-98-01;//“Name of the device model”//
  “type”:CREDIT CARD; //“Card Type used in the transaction”//
  “paymentScheme”: MASTERCARD;//“Issuer Type”//
  “issuerName”: SBI//“Banking Partner”//

This section describes an example of an event logged when a user initiates a transaction after logging into a system. The event captures and stores various key details related to the transaction which are stated below.

• Unique Request ID (*requestId*): A unique identifier for the transaction request, e.g., ca45dt-qur56e-8uyd76-jsT5t6.
• Application Version (*appVersion*): The version of the application being used for the transaction, e.g., 2.0.0.6.
• Application ID (*appId*): The identifier for the application facilitating the transaction, e.g., Tpay.
• User ID (*userId*): A unique identifier for the user, often linked to their registered account or phone number, e.g., 9902496905.
• Event Type (*eventType*): Specifies the type of action performed by the user. Here, TPCLTRNSINI indicates a “Transaction Initiation” event.
• Event Status (*status*): Reflects the outcome of the event, e.g., SUCCESS for a successful operation.
• Event Time (*eventTime*): The timestamp of when the event occurred, e.g., 2021-19-04 19:13:21.579.
• Status Code (*statusCode*): A numerical code representing the status of the event, e.g., 200 for success.
• Trace ID (*traceId*): A unique identifier used to trace the event in logs, e.g., 12674.
• Device Model (*deviceModel*): The model of the device used for the transaction, e.g., Samsung-lf-98-01.
• Card Type (*type*): Indicates the type of card used in the transaction, e.g., CREDIT CARD.
• Payment Scheme (*paymentScheme*): The payment network associated with the card, e.g., MASTERCARD.
• Issuer Name (*issuerName*): The banking partner or card issuer, e.g., SBI.

This detailed event log serves as a record for analyzing transactions, tracing issues, and ensuring system reliability. It captures all relevant user, device, and transaction data to facilitate auditing and debugging if needed.

Various user actions will be recorded in a log file that traverses all “*N*” systems by the system architecture, similar to the preceding Event. We will be able to extract the required output from this log file at various timestamps in terms of the required performance metric.

### 5.3. Hive Queries

To obtain the aforementioned performance metrics, a series of methodical Hive queries are required. A set of Hive queries have been processed to meet the analytics requirements of our proposed system. The various performance metrics mentioned in Section 5.1 are intended to provide the result derivation, and a snapshot of two random Hive queries has been represented below.

Sample Hive-Query:

The following query is for the event “number of active user per month”:

  “Select as Event, substring extract (a month from the cast (event time as timestamp)) as a month, count (userId) as TotalUsers from happy.tnp_prod where event type = ‘TPACCSTATUS' AND ‘select extract Daily Active Users (DAU)' as Event, (a month from the cast (eventTime as timestamp)) as a month, count (distinct userId) as Users from happy.tnp_prod and ‘Daily Transacting Users (DTU) where event type = ‘TPACCSTATUS' userId, count (distinct userId) and ((extract (a month from CAST (eventTime AS TIMESTAMP)))) = (extract (a month from the cast (current_date as Timestamp))) extract (a month from the cast (eventTime as timestamp))) select ‘Transaction per user per month' as Event,a.month, cast ((a.TotalUsers/b.Users) as decimal) and Total from second *b* full outer join first a on b.month = a.month”;

The query analyzes user activity and transaction behavior on a monthly basis from the happy.tnp_prod table. It calculates the *number of active users per month* by counting users associated with the event type TPACCSTATUS and extracting the month from the eventTime field. Additionally, it computes *Daily Active Users (DAU)* and *Daily Transacting Users (DTU)* by counting distinct users engaging daily, filtered by the same event type and grouped by month. Finally, it calculates the average number of transactions per user each month by dividing the total number of active users by the daily user counts, combining results through a join operation. This provides a holistic view of user engagement, activity frequency, and transactional trends for each month.

Similarly, for the event “Number of Active cards,” the Hive query is:

  “Select ‘Active Cards' as Event, enrolled, min (substr (eventTime,1,10)) as Date from jiopay.tpay_rtrs4 where eventstatus = ‘SUCCESS' and event type = ‘TPCRDADD' and event Type = ‘TPCRDRMV' and event status = ‘SUCCESS' enrolid NOT IN (select enrolled as id from jiopay.tpay_rtrs4 where) group by 2) select distinct (Date),count (distinct enrolled) total,Event from first where date> “cast (Date_add (‘Day', −15,cast (current_date as timestamp)) as varchar) group by Event, date”; The query analyzes active cards by tracking events related to card addition (TPCRDADD) and removal (TPCRDRMV) in the jiopay.tpay_rtrs4 table, focusing only on successful transactions (eventstatus = “SUCCESS”). It identifies active cards by excluding those removed, using a subquery to filter out enrolid entries associated with card removal events. For each card, the earliest recorded event date is determined, and the data are grouped by the enrolled ID. Finally, the query calculates the total number of distinct active cards for the past 15 days by filtering events (Date > current_date - 15) and grouping the results by event type and date. This provides a snapshot of card activity trends over a recent time period.

## 6. Experimental Simulation

To implement this setup effectively, the machine configuration used is depicted in Table 2. Also, this section includes a discussion of two input instances (6.2) that is described in Section 5.2. It provides a snapshot of the required parameters about their values.

### 6.1. Machine Configuration

The following table presents the machine configuration for an experimental data processing setup, detailing the resource specifications for each component in the pipeline, including Kafka, Hadoop, NiFi, Presto, and visualization servers. These specifications ensure efficient data ingestion, processing, and analytics, while maintaining scalability and fault tolerance across the system.

### 6.2. Input Instances

This analysis captures test case generated from system configuration as input instances and examines key metrics on user activity and transaction events, including active user counts over five days and detailed records of individual transactions, providing insights into usage patterns and system performance.

#### 6.2.1. Case I

The initial data capture logs a successful transaction event, tracking details like request ID, app version, user ID, event type, status code, and device model for a user completing a MasterCard credit card transaction.

i. Input:
  {
  “request_Id” = ca45dt-qur56e-8uyd76-jsT5t6, “app_Version” = 2.0.0.6, “app_Id” = Tpay, “comp” = PSR, “user_Id” = 9902496905, “user_Type” = N, “event_TYpe” = TPCRDTRMS, “event_Time” = 2021-19-04 19:13:21.579, “status” = SUCCESS, “status_Code” =200, “trace_Id” = 12674, “channelId” = agudw6shhd5, “deviceModel” = samsung-lf-98-01, “type” = CREDIT CARD, “paymentScheme” = MASTERCARD, “issuerName” = SBI
  }
ii. Dashboard and corresponding result

The result has been organized into the dashboards below and summarized using the input above applied log events and queries. With the assistance of the appropriate Hive queries, we can conceptualize a real-world scenario for some of the six performance metrics that have been selected.

**Figure 4:**
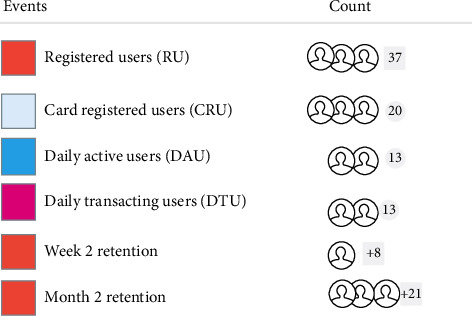
Dashboard screenshot.

The resulted dashboard in Figure 4 displays a clear visualization of all the events that are resulted during the input snapshot. There are 37 registered users, 20 card-registered users, 13 daily active users, and 13 daily transacting users. Similarly, the retention figures for week 2 and month 2 are 6 and 21, respectively.

**Figure 5:**
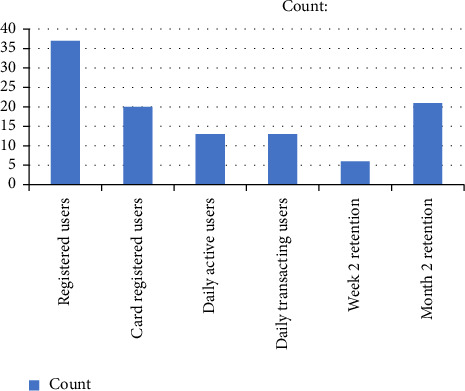
Graphical illustration of the dashboard.

The “Card analytics matrix” depicted in Figure 5 is the “Dashboard Graphical Presentation” for a specific period executed through 6 Hive-queries.

#### 6.2.2. Case II

With additional Hive queries, a second capture of five days' activity for various performance metrics was made. The result is shown below. Here, we have chosen to keep track of user activities for five days, from April 29, 2022, to April 29, 2022. The same-length output screenshot is shown in the table. Number of active users and number of active cards are the two types of active users (events 1 and 3) listed below.

**Figure 6:**
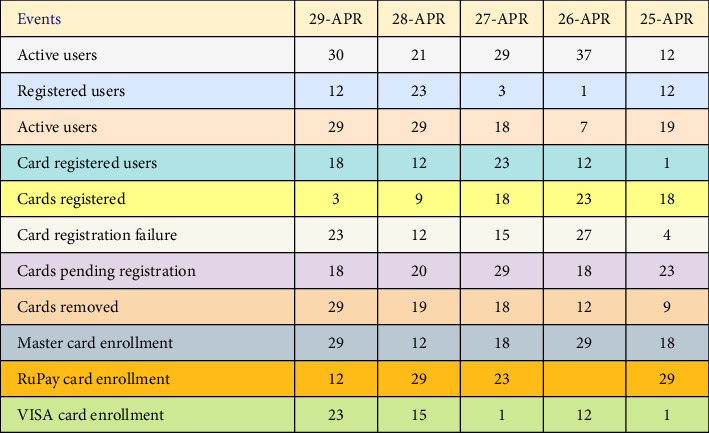
Second case: “card analytics screenshot.”

The above metric analysis of Figure 6 is depicted graphically in the following over time. The duration of various events over the course of five days is shown on the *X*-axis. The event names have been abbreviated in the appropriate locations.

**Figure 7:**
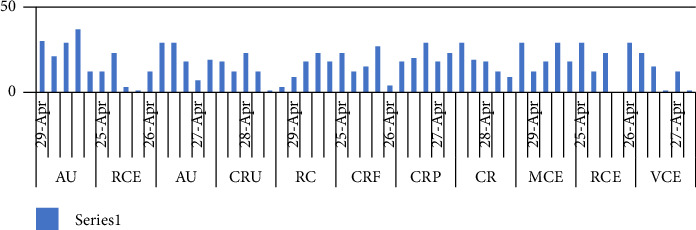
“Graphical card analytics—Case II.”

Figure 7 visually represents the number of instances for each event over a five-day period (April 25, 2022, to April 29, 2022) of the second case (shown in Figure 5).

From the above cases, this study demonstrates the processing and visualization of large-scale data in the system on the basis of insights into user engagement and transaction behavior. Case I illustrates a depth of individual transaction analysis, whereas Case II is a longitudinal trend that spans several days which further builds long-term transactional reports.

### 6.3. Advantages of Hadoop Over Traditional Methods

Hadoop architecture plays a pivotal role in handling the vast amounts of data required for effective performance metric analysis and visualization, as demonstrated in the scenarios outlined above. The system's ability to execute Hive queries, process large-scale log events, and manage data-intensive operations over a distributed framework ensures seamless analysis of key metrics.

i. Data handling and storage:
  HDFS efficiently stores and manages the raw log events and user activity data collected over time. For instance, the five-day activity data for active users and active cards are stored in HDFS, allowing high-volume and structured data to be processed without performance bottlenecks.
ii. Data query and processing:
  Hive, integrated into Hadoop, simplifies querying and analyzing large datasets using SQL-like syntax. By applying Hive queries, the research captures and conceptualizes real-world scenarios for performance metrics such as the number of active users and active cards.
iii. Scalability:
  Hadoop processes large datasets in parallel, ensuring quick execution of queries like the five-day activity analysis for user events.
iv. Handling diverse data types:
  Data found in the banking industry come in three forms: structured (transactions), semistructured (logs and XML files), and unstructured (social media insights or customer feedback). Unlike traditional databases that primarily handle structured data, Hadoop supports and processes all these data types, enabling comprehensive analysis and deeper insights.
v. Cost-effectiveness:
  Although traditional relational databases require expensive hardware for large-scale data storage and processing, Hadoop works perfectly well with commodity hardware, which reduces costs while scaling storage and processing power. This is exactly what big financial institutions are looking for—a highly scalable solution that saves costs without compromising performance.

## 7. Conclusion and Future Scope

This research focuses on the banking sector, demonstrating the unique and effective application of a Hadoop-based architecture, integrating tools like Kafka, NiFi, Presto, and Java, for processing and analyzing large volumes of user activity data from multiple systems. By utilizing input from an industry-based project, we successfully simulated real-world banking scenarios, overcoming the limitations of accessing real-time bank data. The framework enabled the tracking of critical metrics, such as registered users, card-registered users, daily active users, daily transacting users, and retention rates, providing both real-time and historical insights. The results were visualized in dashboards, offering clear representations of user behavior, registration trends, card usage, and event patterns over specified periods. Additionally, a five-day activity analysis provides further insights into user engagement and event occurrences, showcasing the system's capacity to handle diverse and dynamic datasets. The proposed model's ability to efficiently analyze detailed logs enhances operational efficiency, supports data-driven decision making, and provides a scalable solution for tracking and improving banking services. The primary limitation of this model is its reliance on industry-provided project data rather than real-time banking data, which may not fully capture the complexities of live banking systems. However, this adaptable architecture can be adopted by banks or financial institutions to improve customer satisfaction, monitor system performance, and gain valuable business insights in a rapidly evolving digital environment.

## Figures and Tables

**Table 1:** Various events and corresponding purposes.

Name of the event	It is purpose
“TPCRDADD”	When any digital cards are added, the event is created
“TPACCSTATUS”	The status of the account is checked using this event
“TPCLTRNSINI”	When a transaction is started, this is made
“TPCLTRNSUSS”	For a successful transaction, this event is triggered
“TPCLTRNFAILED”	When the transaction fails, this event takes place
“TPACCREG”	For a registered account, this event is made
“TPACCCRESSUC”	For a successful account relation, this event is created
“TPCRDRMV”	When the card is taken away, this event takes place
“TPCRDTRMS”	When a transaction is not finished, this event is triggered

**Table 2:** Machine configuration.

Component	Resource	Specification
1. Kafka cluster	CPU	4–8 cores per node
	Memory	16–32 GB RAM
	Storage	SSD-based storage with a minimum of 500 GB per node for fast read/write operations
	Network	High bandwidth for real-time data streaming
	Cluster size	Minimum of 3 nodes to ensure fault tolerance

2. Hadoop cluster (HDFS + Hive)	CPU	8–16 cores per node for processing tasks
	Memory	32–64 GB RAM per node to handle large datasets and in-memory operations
	Storage	1–5 TB of storage per node, preferably on HDD for storage and SSD for caching
	Cluster size	At least 4 nodes to balance data replication and computational needs

3. NiFi server	CPU	4 cores
	Memory	10 GB RAM
	Storage	200–500 GB (SSD recommended for high-speed data ingestion)

4. Presto and analytics server	CPU	Eight cores
	Memory	32 GB RAM
	Storage	SSD with at least 500 GB for caching and processing
	Network	High-speed network connectivity to access HDFS data and serve queries efficiently

5. Visualization server	CPU	4–8 cores
	Memory	16–32 GB RAM
	Storage	SSD with 200 GB or more for visualization tools and dashboard storage

## Data Availability

We have collected data from an industry-based project.
